# Missing men with tuberculosis: the need to address structural influences and implement targeted and multidimensional interventions

**DOI:** 10.1136/bmjgh-2019-002255

**Published:** 2020-05-04

**Authors:** Jeremiah Chikovore, Madhukar Pai, Katherine Chisholm Horton, Amrita Daftary, Moses Kelly Kumwenda, Graham Hart, Elizabeth Lucy Corbett

**Affiliations:** 1 Human and Social Capabilities Research Division, Human Sciences Research Council, Durban, South Africa; 2 McGill Global Health Programme & McGill International TB Centre, McGill University, Montréal, Ontario, Canada; 3 Department of Clinical Research, London School of Hygiene and Tropical Medicine, London, UK; 4 Department of Infectious Disease Epidemiology, London School of Hygiene and Tropical Medicine, London, UK; 5 Dahdaleh Institute of Global Health Research, York University, Toronto, Ontario, Canada; 6 Centre for the Aids Programme of Research in South Africa (CAPRISA), University of KwaZulu Natal, Durban, South Africa; 7 Malawi-Liverpool Wellcome Trust Clinical Research Programme, Blantyre, Malawi; 8 School of Life and Medical Sciences, University College London, London, UK

**Keywords:** tuberculosis, public health, health services research, health policies and all other topics, epidemiology

## Abstract

Tuberculosis (TB) is treatable but is the leading infectious cause of death worldwide, with men over-represented in some key aspects of the disease burden. Men’s TB epidemiological scenario occurs within a wider public health and historical context, including their prior sidelining in health discussions. Differences are however noticeable in how some Western countries and high TB and HIV burden low and middle-income countries (LMIC) including in Africa have approached the subject(s) of men and health. The former have a comparatively long history of scholarship, and lately are implementing actions targeting men’s health and wellness, both increasingly addressing multilevel social and structural determinants. In contrast, in the latter men have received attention primarily for their sexual practices and role in HIV and AIDS and gender-based violence; moreover, interventions, guided by the public health approach, have stressed short-term, measurable and medical goals. Debates and the limited available empirical literature on men’s engagement with TB-related healthcare are nevertheless indicating need for a shift, within TB work with men in high burden LMICs towards, structural and multicomponent interventions.

**Summary box**

- Despite being treatable, tuberculosis (TB) is the leading infectious cause of death worldwide.
- TB epidemiology reflects systematic inequities including an over-representation of men in some key poor indicators.
- The role of structural and multilevel factors in health status and behaviour, including for men, is increasingly acknowledged particularly in some Western settings.
- In high TB burden low-income settings, men’s health and TB epidemiological situation have largely been overlooked, and men have been focused on mainly for their sexual practices and role in HIV/AIDS and gender-based violence.
- Addressing men’s TB epidemiological situation requires acknowledging the complex determinants and implementing multifaceted interventions.

## Introduction

Tuberculosis (TB) is treatable but is the leading infectious cause of death worldwide with men over-represented in some key aspects of the disease burden.^1^ Globally in 2018, adult men (aged 15 years and older) constituted 55% (vs 31% for women) of TB deaths in HIV-negative people, and 49% (vs 38% for women) of TB deaths in HIV-infected people.^1^ Men also accounted for 57% (vs 32% for women) of people who developed TB.^1^ Analysis of surveys implemented in African and Asian countries over the last two decades yielded a male-to-female (M:F) prevalence ratio of 2.21 (based on 56 surveys) for bacteriologically positive TB, and 2.51 (based on 40 surveys) for smear-positive TB.^2^ Men’s TB situation echoes their disadvantages in several areas of health, including poorer health services utilisation.

## Men’s consistently poor health outcomes and healthcare services underutilisation

Despite gains in global health in recent decades, adult men lag behind women in various health indicators. In 2016, life expectancy surpassed 80 years in 57 and 10 countries for women and men, respectively.^3^ Indicating a widening gap, between 1970 and 2016, life expectancy increased globally by 13.5 years for men and 14.8 years for women, while the gap between men and women increased from 4.2 years to 5.5 years.^4^ Men also have poorer outcomes in HIV and AIDS where they delay testing and starting treatment; in addition, they have worse adherence and higher loss to follow-up when on treatment, and consequently higher chances of dying.^5^ HIV drives TB in high burden countries, and men’s poorer outcomes in HIV and AIDS extend to TB. Table 1 shows sex differences in TB incidence, notification and prevalence. In 2018, the M:F ratio of incident TB cases for all ages in the different WHO regions ranged from 1.3 to 2.0, and the M:F notification ratio was 1.7 globally.^1^ For the period 1970–2016 the age-standardised TB incidence rate per 100 000 people for men was nearly 1.8 times that of women (154.4 vs 86.3), and the TB mortality rate per 100 000 at least twice higher in men than women (21.9 vs 10.8). 6

**Table 1:** M:F sex ratios for incidence, notification and prevalence in selected regions and countries, and from analysis of surveys from different parts of the world; and contributions by sex to global TB notifications, cases and deaths

Sex ratios for incidence, notification and prevalence in selected regions/countries	
*Incidence—all ages (2018*)	*M:F ratio*
WHO Eastern Mediterranean Region	1.3*
WHO European Region	2.0†
WHO West Pacific Region	2.0†
*Notification (2018*)	*M:F ratio*
Global	1.7
WHO Eastern Mediterranean Region	1.1*
WHO West Pacific Region	2.6†
*Prevalence (from analysis of surveys from different parts of the world,1993 - 2016)* ‡	*M:F ratio*
Bacteriologically confirmed TB	2.21
Smear-positive TB	2.51

Contributions by sex to global TB notifications, deaths and cases (2018)	
*Notifications*	*%*
Men	58
Women	34
*Adult TB deaths: HIV*- *uninfected*	*%*
Men	55
Women	31
*Adult TB deaths: HIV infected*	*%*
Men	49
Women	58
*Total TB cases*	*%*
Men	57
Women	32

Source of data, except ‡: WHO.^1^

*Lower value among WHO reporting regions

†Upper value among WHO reporting regions

‡Source of data.^2^

M:F, male-to-female; TB, tuberculosis.

Explanations for the sex differences in TB rates are complex and subject of debate, but tend to include possible differences in biology,^7–9^ in risk-exposing behaviours and occupations,^10^ in transmission dynamics,^11^ or in health services access, use, diagnostic and reporting practices.^1 8^ The accentuation of sex differences after childhood also suggests a role for environmental factors.^1 12^ Additionally, the prevalence-to-notification ratio is held to indicate a gap in diagnosis and reporting,^2^ and greater ratios among men imply women access diagnostic and treatment services more efficiently. Modelling work has further established that these gaps are significant, with delays from disease onset to treatment initiation 1 year longer for men than for women in Vietnam and Malawi.^13^ The sex differences have also persisted despite successes of the WHO-recommended strategy for controlling TB, DOTS (Directly Observed Treatment, Short Course), and interventions against HIV both driving declines in TB, and despite higher HIV prevalence in women in countries with generalised HIV epidemics.^2 10^

Evidence from related HIV literature, and research from around the world consistently reporting men using health services less than women for most illnesses, supports a view of men accessing TB-related health services poorly. Among patients registered with general practice in the UK primary care, men’s crude consultation rates were found to be 32% lower than for women.^14^ In Europe, sex differences in use of primary care services ranged from approximately 5% in the Czech Republic and Austria to approximately 18% in Cyprus and Greece.^15^ However, some argue women may be accessing services more but only during the reproductive years.^16^ Women’s excess consultation for general health matters has also been questioned for relying on self-reported survey data and paying limited attention to underlying morbidity.^14^ For TB, it has also been suggested that while men delay seeking care, women face greater diagnostic barriers because they contact healthcare services more frequently, but reaching more easily accessible yet less qualified providers, and for symptoms not specific to TB.^17^

## Men in public health research and policy

Men have traditionally been sidelined in public health due, in part, to early binary classification of sexes by biological attributes, which meant women were exclusively associated with reproduction and hence targeted in family planning and reproductive health research and policy.^18^ Later realisation that gender power and relational dynamics influenced the success of the programmes prompted efforts to include men.^18^ Towards the end of the last century, the unfolding HIV and AIDS epidemic particularly in Africa began to draw interest in men for their sexual practices, which were seen as fuelling disease. However, even when men were included in family planning, sexual and reproductive health, and HIV and AIDS efforts, the primary beneficiaries were still considered to be women.

Differences are observable in how some Western countries and low and middle-income countries (LMIC), particularly the high TB and HIV burden, have handled the subject(s) of men and health. In some Western countries, men, masculinity and health have been the focus of fairly considerable scholarly work over the last odd half century. During this time, analyses generally moved from positivist and essentialist concepts (which stress uniformity within genders, and the role of biological or innate psychological traits in behaviour) towards ecological and social constructionist concepts (which consider historical and contemporary forces, context, and race, class and gender relations in explaining behaviour).^19–21^ Being broad and encompassing, social constructionism and related constructs have in recent decades informed substantial research on men’s behaviours and health. Specific topics where they have been applied in the West include managerial settings,^22 23^ schooling and boys’ achievement^24^ and participation in activities such as dance,^25^ perceptions of women in soccer,^26^ HIV and AIDS, and specific male diseases such as prostrate and testicular cancer.

On the African continent, on the other hand, masculinity debates are relatively incipient. A few countries, principally South Africa, have had a longer scholarly history, addressing mainly men’s sexuality and risk-taking behaviours, and gender-based and other forms of violence. Some of the earlier research aimed to document men’s behaviour and attitudes, generally using assumptions of linear causation.^27^ From around the turn of the century, masculinity scholarship increased rapidly, the bulk now also influenced by ecological frameworks. This work has discussed the role of *apartheid* and colonial administration-related labour migration and mining economies, breakdown in social and communal relationships, and institutions such as factories, mines, prisons, farms and schools in cultivating various versions of masculinities.^28–36^

Policies and interventions appear to have lagged behind globally, although lately some Western countries seem to be making greater progress compared with LMICs including in Africa. In the USA, concern was raised as early as the ‘60s regarding the silence about men’s health in public conversations, including presidential campaigns, despite their worse life expectancy and engagement with health services.^37^ Initiatives on men’s health in some Western countries only took off around the turn of the century, often driven at regional or local level, or by non-government organisations.^38^ Those by central governments struggled to survive, partly because they were interrupted when administrations changed.^38^

What is believed to be the world’s first policy on men’s health was thus only published by Ireland in 2009, with countries such as Australia—with a long history of engaging with men’s health issues—also making noticeable progress.^38^ Presently, few countries (namely Ireland, Brazil, Iran and Australia) have national health policies or strategies specifically addressing men.^39^ Canada has formally identified gender equity as a goal of health policy, and England and Wales built momentum from the establishment of the Men’s Health Forum by the Royal College of Nursing in 1994.^38^ The first specialist university department on men’s health was established at Leeds Metropolitan University in 2004, and in Scotland, the first primary care centre dedicated to improving men’s health was set up in 2001. National Men’s Health weeks, men’s health organisations (namely the European Men’s Health Forum, Nordic Men’s Health Network, Men’s Health Network in the USA, and Health Advice for Australian Men, and Men’s Health, in Australia), marathon events and conferences are being implemented across the Western world, although some countries are trailing behind.^38^ In 2011, the European Union commissioned *The State of Men’s Health in Europe* report, and recently, the region introduced a men’s health strategy, which focuses on social determinants of health and advocates for gender responsive health services.^40^

Women’s heightened social, economic, and health vulnerability, limited health access, and power disadvantage, which take unique forms in high TB and HIV burden LMICs, have seen efforts there being directed towards women-centred initiatives, and gender vulnerability being equated with women in TB research and policy.^41–51^ In these settings, particularly in parts of Africa where HIV drives TB, men have received attention mainly in relation to their sexual practices and role in the related HIV and AIDS epidemic and gender-based violence. Services for HIV and AIDS were also initially linked to maternal and child health, 52 with the result, for men, attending health services was experienced as stigmatising. Implementation activities in these settings also tended to pursue quantitatively measurable, short-term or primarily clinical outcomes.^53^ They additionally sought to change men’s attitudes through activities such as mass media campaigns, education, community workshops, engagement of traditional leaders^54 55^ and involvement of men in participatory activist fora.^56^

## Why do men not engage with healthcare services?

A diverse literature that straddles qualitative, survey, modelling, and non-fiction work, different world regions and several decades, and various social and health topics from a range of theoretical positions, highlights key determinants of men’s use of health services. The determinants can be (re)framed within gender relational theory, an approach within the social constructionist paradigm and which acknowledges how women and femininity, and men and masculinity shape each other and society at large, and simultaneously locates gender relations within wider social, political, historical and economic contexts.^20 57^

The present-day layout, staffing and interaction of primary health facilities with men can be seen as part remnant of how primary healthcare was historically modelled around maternal and child health. Men report feeling alienated at primary care facilities 58 59 which are normatively considered places for women and children^60^ but also felt as not serving men in sensitive and empathetic ways. Men also fear opportunity losses should they pursue activities such as seeking healthcare, which in their view may not have evident or immediate economic value. Anxieties about being publicly chastised at health facilities and blamed for causing their own illness and that of women related to them are further deterrents.^56^

Outside the healthcare system, men are deterred from seeking healthcare because this behaviour is experienced or seen as a weakness. This is significant where men who display lack of control face ridicule and social devaluation from peers, women and even children.^61 62^ Men thus consciously or unwittingly suppress illness avoiding or compensating for appearing to be feminine or not in control. One way they do this is by deeming themselves naturally stronger physically than women.^63 64^ They also overlook illness while striving to fulfil duties of providing for and inspiring families, a responsibility many nevertheless increasingly find hard to achieve.^63 65^

On the African continent the male breadwinner role emerged as a creation from the colonial past,^66^ with the entire colonial process resulting in ‘missing men who occupy marginal positions within households and communities’^67^ (p 19). In South Africa the segregationist policy of *apartheid* dispossessed non-White communities of land, and their labour was appropriated over decades while they were condemned to humiliatingly bleak lifetime opportunities. Movement and residence restrictions resulted in difficult living conditions for families, and communities in which poor social connectedness, unemployment, ethnic tensions, exchange sex and gangsterism prevailed. Selective application of justice and policing brewed resentment and violence within communities and against the system. Many men, on whom the expectation to earn cash had fallen, struggled to raise money to meet their provider roles or other status-conferring needs such as marriage.

Failure to achieve the socially valued masculine grade prompts in men intensified efforts to demonstrate possession of the attributes, or a resort to more accessible versions, and pursuit of these *in extremis* no matter the health implications.^68^ A systematic review on healthcare seeking for depression found that, in addition to engaging in other ‘escape’ behaviours, namely risk-taking, anger-fuelled conflict, and increased work hours, men used short-term strategies of turning to substance and alcohol use.^69^ They additionally concealed distress, re-emphasised strength and resorted to performing masculinity publicly or adhering to traditional concepts of masculinity.^69^ Depression, alcoholism, danger and death thus became common in non-White communities in South Africa^62^ and more widely across colonial Southern Africa’s mining and urban communities,^28^ and formed the context in which men leant to face and inflict danger, and hence also to suppress or overlook it.

## Why do men not engage with TB care?

The prior focus on women in TB research, policy and funding has meant little research exists on men’s TB care access barriers, and most of this has come out of the African continent. This literature, together with related studies on HIV, gender and masculinity, and healthcare seeking illuminates men’s TB healthcare access barriers. Some barriers seem or are indeed similar for men and women: it is nevertheless critical to understand from a gendered lens whether and how they have different implications for different genders.

Fear and anxiety are, for example, well reported with regard to engagement with TB (and HIV) care, but assume nuanced forms through a masculinity-mediated lens. Men are reported in studies delaying seeking healthcare for cough due to fear, or avoiding a possible diagnosis, of TB which is considered ‘serious’ or confirmatory of HIV.^60 70–72^ The link between cough, TB, HIV and highly-likely death in high HIV burden settings fuels stigma of cough and TB at community and family levels.^1 73–75^ Resulting fears,^70 76^ while affecting both women and men, take special forms when they intersect with masculinity expectations for men.

An expectation to display control over their lives explains part of men’s fears and anxieties. TB is perceived and experienced as a virulent disease that hampers independent functioning, and whose treatment drains financial resources. 63 65 71 76 In turn, this interferes with socialisation, alcohol use and sexual activity^60 70 71^ accentuating anxieties about being investigated and/or confirmed with disease. Similar threats to socially valued concepts of masculinity are reported for HIV and AIDS.^34 77–79^

Men also fear being seen at primary care facilities, whose association with women potentially attracts such unwanted labels as being ‘lesser than men’ or having an exceptional illness.^60^ They thus regularly infer about their own health from outcomes of investigations on their women spouses, who also have more connection to primary care and HIV testing services.^80^

Anxieties even where health services are available free of charge, and when antiretroviral therapy has improved longevity and quality of life with TB or HIV, testify to the complex dynamics surrounding men’s TB-related healthcare seeking. Men must deal with unemployment or insecure or low-paying jobs, contend with erosion of male privilege from efforts to promote gender equality, and manage various intersecting forms of marginalisation in their daily lives.^61 65^ Scarce employment and limited job security mean, for example, those who display signs of ill health or are confirmed with TB are at risk of losing their jobs.^65 76^ Simultaneously society, other men and women put pressure on men to display strength, good health, and self-sufficiency, and ability to provide basic and consumer needs to their families.^63 65^ Being ill and physically constrained, or merely seeking healthcare, or acknowledging minor or early-stage illness can thus entail a special form of stigma for men.^63 65^ Furthermore, drug, transport and food costs while seeking care for TB in LMICs,^74 76^ exacerbated when health system challenges necessitate multiple visits, while not specific to men may compel them to relegate healthcare in favour of livelihood needs.

Those undertaking to be investigated encounter at health facilities long waiting times or opening hours unaccommodative of their schedules,^70 72^ or jostle in queues with women and children for attention.^76^ Delays at healthcare facilities affect men differently where they are designated primary breadwinner, employment is scarce with limited social protection, and any time away from ‘working’ means huge opportunity costs. Spending time waiting to be served may be experienced as demeaning by men, who must consistently publicly demonstrate active pursuit of earning opportunities. These experiences for men are worsened by frustrated and overworked health services staff acting discourteously, communicating poorly about algorithms and belittling or blaming men for allegedly causing themselves to be ill and burdening health systems.^60 65 72^ Unfriendly healthcare services are widely reported and affect all groups of patients. Still, public humiliation likely elicits specific feelings in men, who visit health facilities less and would feel emasculated before women and children, whom they ordinarily seek to lead and protect.

These complexly intersecting factors trigger responses to illness such as waiting until symptoms are unbearable or life-threatening. Waiting can entail outright avoidance or putting off of healthcare seeking^60 63 69^; or pursuit of distractions, possibly ones that reaffirm valued masculine traits, like working intensively or socialising and drinking excessively to fend off illness.^60 63^ Studies have described men’s preference for using traditional medicine and alternative forms of treatment, and for seeking healthcare first from places other than where they can be diagnosed formally.^74 75 80^ In avoiding healthcare, men may use socially appropriate alibis such as being unable to spare time owing to work demands.^65^ Illness responses often taken as evidence of men’s stoicism and irresponsibility are likely outcomes of interactional dynamics involving structural factors and social roles. Men’s role as bread earners also forces some to seek ‘fast cures’ from sources, often private (eg, drugstores, unqualified practitioners, traditional healers) that offer highly variable or poor quality care.^81^

## Finding men with TB who are missed by programmes

Management of TB (and HIV and AIDS) in LMICs has relied on WHO’s public health approach.^82^ This approach stresses standardised and simplified treatment protocols, decentralised service delivery to reach large numbers of people through primarily the public sector, and working with lower level healthcare workers to deliver care,^82^ and in some programmes involves backing of free healthcare services to the public. However, the historical and more recent declines in TB burden largely happened alongside improvements in socioeconomic indicators.^83^ The End TB strategy,^84^ Moscow Declaration to End TB, and United Nations High-Level Meeting (UNHLM) on TB are among the growing voices advocating for anti-TB efforts that fall within the broader sustainable development agenda, tackle multidimensional determinants of health, strengthen health services delivery, enhance private sector engagement and quality and move to universal healthcare and active case finding, among others.^1 85^

In high TB and HIV burden LMICs, many men’s health-related behaviours and outlook are engendered within structurally conditioned parameters. The complex influences particularly on their TB-related healthcare seeking call for analyses and interventions that consider in context men’s role expectations, means available for pursuing these, and implications of failure or its threat. Structural conditions and gendered interactional dynamics imply men are discouraged from displaying weakness and acknowledging illness, face opportunity and real costs from illness incapacitation and healthcare seeking, and encounter unaccommodating and emasculating health services. Interventions, guided by ecological and intersectionality frameworks, need to straddle policies at international and national levels, structural and social actions at local, national, societal and community levels, and behaviour and knowledge at interpersonal and personal microlevels. (For an illustrative draft intervention framework, see ref 86.)

Exemplifying high-level global action, in 2018 the UNHLM on TB called for recognising the higher TB prevalence in men. In the same year, the WHO advocated for enhancing men’s access to and use of health services,^87^ while the Special Programme for Research and Training in Tropical Diseases revealed plans to expand training and research support on gender, and held a meeting of experts with masculinities and TB on the agenda. In its recent annual meetings, the Union World Conference on Lung Health has also featured presentations and seminars on men and TB. On the HIV and AIDS front, the International AIDS Society included several sessions and a preconference forum on men at its 2019 scientific conference.

Beyond developments taking place internationally, interventions to engage men in TB need to include social protection mechanisms, promotion of wellness in and through men’s different work settings^40^ and awareness promotion including highlighting the importance of early diagnosis and treatment.^88^ Equally, the preparedness of public and private healthcare services to accommodate men (and women) based on their roles, circumstances and needs—in line with the ethos of client-centred care^85^—should be evaluated and supported. Engagement of private and informal sectors, in particular improvement of quality of TB care in these sectors, is critical to helping find missing patients with TB including men.^89 90^ Flexible opening times, mobile services, algorithms reducing waiting times, male-friendly spatial arrangements and healthcare staff trained in male sensitivity are all potential interventions to explore or implemented as suits context.^86^ Examples of tailored interventions that have indicated success include workshops for men implemented as part of the gender-based violence response,^91^ and reaching men in HIV testing trials through settings such as bars, churches and workplaces, and using mobile testing services.^92^ These strategies fit well demands of men’s TB epidemiological situation and can be adapted while retaining sight on the value of multieffect and synergistic actions to address complexities of men’s TB care access.

## Conclusion

Men’s disproportionate TB epidemiological burden and poor healthcare access reflect a broader pattern of suboptimal engagement with health services, which is barely helped by their absence in funding and policy. Greater attention to men, with research and interventions addressing the range and complexity of their TB and general healthcare engagement determinants, and supported by robust social science research to illuminate contextual and relational dynamics, is needed. This does not denote turning attention from women or seeing them as not vulnerable: women’s special vulnerability to TB and social and structural injustices^93^ is well documented and necessitates their continued prioritisation. Rather, it reminds how gender affects women and men and their experiences of disease predominantly differently, with wider public health ramifications.
